# Dynamic analysis of agricultural carbon emissions efficiency in Chinese provinces along the Belt and Road

**DOI:** 10.1371/journal.pone.0228223

**Published:** 2020-02-07

**Authors:** Hua Zhang, Sidai Guo, Yubing Qian, Yan Liu, Chengpeng Lu

**Affiliations:** 1 Sichuan Circular Economy Research Center, Southwest University of Science and Technology, Mianyang, China; 2 School of Public Administration, University of Electronic Science and Technology of China, Chengdu, China; 3 Institute of County Economy Development & Rural Revitalization Strategy, Lanzhou University, Lanzhou, China; 4 Key Lab of Pollution Ecology and Environmental Engineering, Institute of Applied Ecology, Chinese Academy of Sciences, Shenyang, China; 5 Key Lab for Environmental Computation and Sustainability of Liaoning Province, Shenyang, China; Institute for Advanced Sustainability Studies, GERMANY

## Abstract

To better understand the agricultural resources and environmental problems of the provinces along The Belt and Road in China, it is critical to investigate their agricultural carbon emission efficiency and evolutionary trends. Based on the panel data of 18 key provinces and cities between 2006 and 2015, this paper evaluated the agricultural carbon emission efficiency with the data envelopment analysis–Malmquist model and further explored their dynamic evolutionary trends. There were several main findings. First, the efficiency levels of agricultural carbon emissions showed significant regional differentiation among the areas, with that along the 21st-Century Maritime Silk Road being much higher than that along the Silk Road Economic Belt. Second, technical efficiency was the key factor that restricted the improvement of the comprehensive efficiency of agricultural carbon. Third, most provinces invested in too many redundant and unreasonably allocated resources, showing a trend of diminishing returns to scale. Last, According to dynamic evolution analysis, the total productivity still demonstrated a diminishing trend. This paper provides some suggestions for effectively improve the efficiency of agricultural carbon emissions in China, such as optimize the agricultural industrial structure, increasing the investment of carbon emission reduction technology, and implementing a carbon emission quota clearing system. This paper contributes to the improvement of the environment in China.

## Introduction

Global warming involves the essential task of worldwide environmental governance. The Fifth Assessment Report of the Intergovernmental Panel on Climate Change (IPCC) indicated that the global temperature has raised by 0.85 °C from 1880 to 2012, and the increase in surface temperature every 10 years is higher than that of the prior 10-year period [1]. Global warming has triggered glacier melting, sea level rise, deterioration of freshwater resources, the slow speed of air flow, and the formation of haze, all of which also affect the survival and development of human beings. Therefore, solving the problem of global warming is critical for the long-term stability and well-being of the earth. By 2400, the existing atmospheric greenhouse gas (GHG) components will increase the global temperature by 1 °C on average, and new GHG emissions will produce an additional increase of 2 to 6 °C, resulting in a 10–25-cm rise in the sea level per century. Carbon emission is one of the primary sources of GHGs, and therefore a reduction in carbon emissions has become the principal means of global governance of GHGs. Since the industrial revolution, the massive exploitation and use of fossil energy have rapidly increased industrial carbon emissions [2]. However, the carbon emissions of agricultural activities, such as agricultural land use, crop planting, and livestock farming, cannot be ignored. Given widespread agricultural activities, agriculture has become an important source of GHG emissions [3, 4] while being the most vulnerable to climate change [5, 6]. Therefore, many scholars have extensively researched carbon dioxide emissions from agriculture [7, 8].

The issue of agricultural carbon emissions has been investigated by researchers from various perspectives based on different countries and regions, such as the causality of agricultural CO_2_ emissions [9–13]. Samuel and Phebe investigated the relationship between CO_2_ and agriculture in Ghana between 1961 and 2012, their findings showed that CO_2_ emissions affect the production of coarse grain, cocoa bean, fruits, and vegetables; they suggested the integration of climate change measures into Ghana’s national strategies to strengthen the country’s effort to achieve sustainability [10, 14]. Some scholars studied the relationship between carbon dioxide and economic growth in China’s agricultural sector using the carbon dioxide decoupling theory [15, 16], investigating the structure and features of agricultural production from 1993 to 2013 in Shanghai and measuring the increase in agricultural carbon emissions, and suggested that improving energy efficiency and reducing the amount of energy consumption will be possible solutions for further emissions reduction during agricultural development. Luo et al. stated that, “fertilizer, in-season rice cultivation, and cattle generated the most CO_2_ emissions in the categories of agricultural production activities, farming, and livestock husbandry, respectively.”, and also explored the spatial-temporal characteristics of CO_2_ emissions and their intensity in China’s agricultural sector [17].

The carbon footprint of crop cultivation patterns and animal husbandry forms and the carbon balance in the agroecosystem are key topics, such as in the fields of food crops, animal husbandry, vegetables, and fruits during climate change [18–20]. Scholars have estimated the carbon footprint of some products to comprehensively assess agricultural carbon emissions [21–26], and this has provided a reliable data foundation for formulating effective carbon dioxide reduction strategies. Fargione et al. [27] and Arevalo et al. [28] researched the change in the carbon effect in two cases of reclaiming biomass energy crops and transforming agricultural land into fast-growing shortcut woody crops, respectively. Lal suggested that soil erosion is the most widespread form of soil degradation and indicated that soil erosion has a significant impact on the global carbon cycle, and this issue must be considered when assessing the global carbon budget [29]. Kindler et al. found that loss of soil carbon through leaching significantly affects the carbon balance of the agricultural system [30].

The many studies that have been conducted to investigate the critical characteristics of agricultural carbon dioxide emissions not only enriched the carbon emission research system but also laid a solid foundation for the development of this study. However, some research gaps exist and need to be filled in: (1) Most of the related literature is based on a single perspective, lacking the necessary macro coordination. Few scholars have systematically analyzed and evaluated the agricultural carbon emission efficiency of the provinces along the B&R areas. (2) Most authors studied carbon emission efficiency from a macro level or focused on the technical perspective [31–35]. Few scholars have discussed the situation, evolution trend, and convergence of agricultural carbon emission efficiency. (3) From the data perspective, the cross-section data from one year are usually selected as a comparison point, whereas few scholars have used panel data to explore the dynamic distribution and evolutionary trends of agricultural carbon emission efficiency. The paper aims to fill these research gaps. As China is in a critical period of transformation in its agricultural economy, agricultural carbon emissions, as an essential part of China’s carbon emissions, should be included in the government’s energy saving and emission reduction work. The B&R initiative represents a bright new aspect of China’s opening to the world. Efficient measurement of the agricultural carbon emission efficiency in the provinces along the B&R can provide the necessary theoretical basis for the formulation of various agricultural carbon emission reduction policies.

Since its reform and opening, China has made remarkable economic achievements, but rapid growth inevitably results in a massive amount of consumption of resources, making China the largest emitter of carbon in the world [36]. Determining how to effectively reduce carbon emissions has become one of the hot spots for academia in China. In September and October 2013, Chinese Government introduced the Silk Road Economic Belt and the 21st-Century Maritime Silk Road policies. These two strategies are combined as The Belt and Road (B&R) in this study. This B&R development strategy has attracted considerable attention from the international community. In 2017, the amount of trade between China and the countries along the B&R reached 7.4 trillion Chinese Yuan (CNY) and increased by 17.8% over 2016[37], which shows that economic and trade cooperation has produced remarkable results. In the new B&R scheme, China and the countries involved are completing agricultural connections under a dual or multilateral cooperation mechanism. The field of cooperation has been expanding, and the economic benefit of agriculture is increasing in some countries. The total agricultural output value along the B&R reached 2916.72 billion yuan in 2017, accounting for 7.59% of the gross domestic product (GDP). In particular, the total agricultural output value reached 1990.15 billion yuan in the Silk Road Economic Belt, accounting for 11.32% of GDP [38]. In addition, the proportion of agricultural land area is high, but the per capita cultivated land area is lower. This shows that agriculture generally occupies a high proportion in the national economic composition along the B&R and plays a particularly important role in the development of the national economy. With the introduction of policies such as “Guidance on Promoting Green Belt and Road” and “The Belt and Road Ecological and Environmental Cooperation Plan,” the research on agricultural carbon emissions is conducive to strengthening the protection of regional agricultural ecological security and improving the ecological environment of regional agricultural production. Therefore, to understand the agricultural resources and environmental problems of the provinces along the B&R in China, it is critical to investigate their agricultural carbon emission efficiency and evolutionary trends, which provide an essential reference and suggestions to formulate an agricultural carbon emissions reduction policy.

Based on the panel data of 18 key provinces and cities in China between 2006 and 2015, including agricultural input, expected output and unexpected output, we evaluated agricultural carbon emission efficiency with a data envelopment analysis (DEA)-Malmquist model and further explored their dynamic evolutionary trends.

## Methods and data source

### Models

#### Data envelopment analysis model

The data envelopment analysis (DEA) model was developed to help compare the relative performance of decision-making units [39]. As a nonparametric estimation method, DEA is a quantitative analysis method that is used to evaluate the relative effectiveness of different sectors based on the linear programming method, which is based on multiple input and output indicators [40]. DEA generates an efficiency score between 0 and 1 for each of the units, indicating how effectively they are at managing their resources [41]. Charnes et al. [39] proposed the CCR model, and Banker et al. [42] further developed CCR into the BCC model by changing the assumption that the scale returns would change. Many studies have been conducted to analyze carbon emissions using the DEA model [43–46]. Under the assumption of stable returns of scale, the input–BCC model is formulated as follows:

Consider a set of *n* DMUs that is associated with *m* inputs and *s* outputs. Each *DMU*_*j*_ *(j = 1*,*2*, *⋯*, *n)* consumes amount *X*_*ij*_ *(>0)* of input ***i*** and produces amount *Y*_*ij*_ *(>0)* of output ***r***. Consider the observed input *X*_*j*_ = (*X*_*1j*_, *X*_*2j*_, *⋯*, *Xmj)*^*T*^*≥0*, *X*_*j*_*≠0*, and the observed input *Y*_*j*_ = (*Y*_*1j*_, *Y*_*2j*_, *⋯*, *Ymj)*^*T*^*≥0*, *X*_*j*_*≠0* for *DMU*_*j*_.

Generally, the Production Possibility Set (*PPS*) of the BCC model and the corresponding variable returns to scale can be defined as follows:

$$T=\{\left(X,Y\right)|X\geq \sum_{j=1}^{n}{\;\lambda }_{j}X_{j},Y\leq \sum_{j=1}^{n}\lambda Y_{j},\sum_{j=1}^{n}\lambda_{j}=1,\lambda_{j}\geq 0,\forall j\}$$ (1)

To evaluate the efficiency of *DMU*_*j0*_ related to the Production Possibility Set *t*, the linear programming model can be defined as follows:

$$Min\theta =V_{D}$$

$$s.t.\sum_{j=1}^{n}\lambda_{j}X_{j}\leq \theta X_{j0}$$ (2)

$$\sum_{j=1}^{n}\lambda_{j}Y_{j}\geq Y_{j0}\;{(\lambda }_{j}\geq 0,j=1,2\ldots ,n),$$

where $\sum_{j=1}^{n}{\;\lambda }_{j}X_{j}$ is the input factor of this *DMU*, $\sum_{j=1}^{n}\lambda Y_{j}$ is the output factor, and *λ*_*j*_ stands for the weight. The model can be used to evaluate the relative efficiency of *DMU*_*j0*_. Eq (2) uses as few input factors (minθ, $\sum_{j=1}^{n}{\;\lambda }_{j}X_{j}\leq {\theta X}_{j}$) as possible under the condition of $\sum_{j=1}^{n}\lambda Y_{j}\geq Y_{j0}$. When *θ* = 1, the input cannot be reduced since the output does not decrease through the weight combination, and the *DMU* is effective at this time. Otherwise, there is a fictitious *DMU (θ <* 1); that is, the same or even more outputs can be achieved with less input.

After introducing the relaxation variables *S*^−^, *S*^−^ and non-Archimedes infinity *ε*, Eq (2) is transformed into a CCR model.

$$Min[\theta -\varepsilon (E_{1}^{T}S^{-}+E_{2}^{T}S^{+})]=V_{D}$$

$$s.t.\sum_{j=1}^{n}\lambda_{j}X_{j}+S^{-}=\theta X_{j0}$$

$$\sum_{j=1}^{n}\lambda_{j}Y_{j}-S^{+}=Y_{j0}$$

$$\lambda_{j}\geq 0,\;j=1,2,\ldots ,n$$

$$S^{-}\geq 0,S^{+}\geq 0$$ (3)

where *E*_*1*_^*T*^ and *E*_*2*_^*T*^ are m-dimension and s-dimension unit vectors respectively, and non-Archimedes infinity ε is a value less than any positive number but greater than 0. Let λ*, S*^−^, S*^+^, and θ* represent an optimal solution for the CCR model, and the decision theorem of DEA validity is as follows:

If θ* = 1, then *DMU*_*j0*_ is weakly valid.

If θ* = 1, S*^−^ = 0 *and* S*^+^ = 0, then *DMU*_*j0*_ is valid.

To evaluate the pure technical efficiency of regional agricultural carbon emission, the BCC model with non-Archimedes infinity ε is as follows:

$$Min[\theta -\varepsilon (E_{1}^{T}S^{-}+E_{2}^{T}S^{+})]=V_{D}$$

$$s.t.\sum_{j=1}^{n}\lambda_{j}X_{j}+S^{-}=\theta X_{j0}$$

$$\sum_{j=1}^{n}\lambda_{j}Y_{j}-S^{+}=Y_{j0}$$

$$\sum_{j=1}^{n}\lambda_{j}=1$$

$$\lambda_{j}\geq 0,j=1,2,\ldots ,n$$

$$S^{-}\geq 0,S^{+}\geq 0$$ (4)

Compared with the CCR model, the BCC model has only one more constraint $\sum_{j=1}^{n}\lambda_{j}=1$. Let λ*, S*^−^, S*^+^, and θ* represent an optimal solution for the BCC model, and the decision theorem of DEA validity is as follows:

If θ* = 1, then *DMU*_*j0*_ is weakly valid.

If θ* = 1, S*^−^ = 0 and S*^+^ = 0, then *DMU*_*j0*_ is valid.

The BCC model clearly distinguishes the comprehensive technical efficiency (*CTE*), pure technical efficiency (*PTE*), and scale efficiency (*SE*) of each decision-making unit. *CTE* is used to measure the ability of maximum output given a fixed investment or minimum input given a fixed outcome. *SE* is used to measure the extent to which economies of scale can function when the scale reaches an effective point. *PTE* is used to measure the efficiency level after eliminating the scale factor in the comprehensive technical efficiency [47]. Therefore, for each decision-making unit, *CTE* is a combination of *PTE* and *SE*.

#### Malmquist index model

The Malmquist index was first introduced by Malmquist in 1953, and it was initially used to analyze the movement effect of consumption bundles on different indifference curves. Later, this index was applied to production analysis and the measurement of productivity changes in two successive periods [48–50], combining the nonparametric linear programming method and the DEA method, and then the Malmquist index was gradually widely applied.

The distance function is the basis of Malmquist index. It is a method of studying multiple inputs and outputs without any assumptions about producer behavior. The distance function can mathematically be expressed as the reciprocal of the optimal solution of the CCR model and BCC model.

$$D_{i}^{t}(x_{i}^{t},y_{i}^{t})={F_{t}(y_{i}^{t},x_{i}^{t}|C,S)}^{-1}$$ (5)

$$D_{i}^{t}(x_{i}^{t},y_{i}^{t})={F_{t}(y_{i}^{t},x_{i}^{t}|V,S)}^{-1},$$ (6)

where $x_{i}^{t},y_{i}^{t}$ stands for the input and output vector of region i at time t, respectively; S stands for the possible production set at time t; C and V stand for the constant returns to scale and variable returns to scale, respectively; and $D_{i}^{t}(x_{i}^{t},y_{i}^{t})$ represents the distance functions, taking technology standard at time t. The higher the value of $D_{i}^{t}(x_{i}^{t},y_{i}^{t})$, the more effective the production is and the higher the technical efficiency is.

Since the Malmquist index is defined by benchmark technology, the output-based Malmquist index referring to technology at time *t* is as follows:

$$M_{i}^{t}(x_{i}^{t},y_{i}^{t},x_{i}^{t+1},y_{i}^{t+1})=D_{i}^{t}(x_{i}^{t+1},y_{i}^{t+1})/D_{i}^{t}(x_{i}^{t},y_{i}^{t})$$ (7)

Similarly, the output-based Malmquist index referring to technology at time *t+1* is as follows:

$$M_{i,t+1}(x_{i}^{t},y_{i}^{t},x_{i}^{t+1},y_{i}^{t+1})=D_{i}^{t+1}(x_{i}^{t+1},y_{i}^{t+1})/D_{i}^{t+1}(x_{i}^{t},y_{i}^{t})$$ (8)

The Malmquist production index can be defined as the geometric average of Eqs (7) and (8) according to the ideal index construction method [43].

$$M_{i,t+1}(x_{i}^{t},y_{i}^{t},x_{i}^{t+1},y_{i}^{t+1})={\left[\frac{D_{i}^{t}(x_{i}^{t+1},y_{i}^{t+1})}{D_{i}^{t}(x_{i}^{t},y_{i}^{t})}\times \frac{D_{i}^{t+1}(x_{i}^{t+1},y_{i}^{t+1})}{D_{i}^{t+1}(x_{i}^{t},y_{i}^{t})}\right)}^{1/2}$$ (9)

$$M_{i,t+1}({x_{i}}^{t},{y_{i}}^{t},{x_{i}}^{t+1},{y_{i}}^{t+1})=\frac{{D_{i}}^{t+1}({x_{i}}^{t+1},{y_{i}}^{t+1})}{{D_{i}}^{t}({x_{i}}^{t},{y_{i}}^{t})}[\frac{{D_{i}}^{t}({x_{i}}^{t},{y_{i}}^{t})}{{D_{i}}^{t+1}({x_{i}}^{t},{y_{i}}^{t})}\times \frac{{D_{i}}^{t}({x_{i}}^{t+1},{y_{i}}^{t+1})}{{D_{i}}^{t+1}({x_{i}}^{t+1},{y_{i}}^{t+1})}{]}^{\frac{1}{2}},$$ (10)

where *x*_*i*_^*t*^ and *x*_*i*_^*t* + 1^ stand for the input vector of region *i* at times *t* and *t* + 1, respectively; *y*_*i*_^*t*^ and *y*_*i*_^*t* + 1^ stand for the output vector of region *i* at times *t* and *t* + 1, respectively; and *D*_*i*_^*t*^ (*x*_*i*_^*t*^, *y*_*i*_^*t*^) and *D*_*i*_^*t*^ + 1 (*x*_*i*_^*t* + 1^, y_i_^t + 1^) represent the distance functions taking technology standard at times *t* and *t* + 1, respectively, as reference points. Eq (10) is the deformation of Eq (9), which refers to the separation of technological change from technical efficiency change. $\frac{{D_{i}}^{t+1}({x_{i}}^{t+1},{y_{i}}^{t+1})}{{D_{i}}^{t}({x_{i}}^{t},{y_{i}}^{t})}$ is the technical efficiency change from time *t* to time *t* + 1, and the rest is *TC*, which is the technical change from time *t* to time *t* + 1.

$$\begin{array}{l} M_{v,c}{}^{t,t+1}=\\ \frac{D_{v}{}^{t+1}\left(x_{i}{}^{t+1},y_{i}{}^{t+1}\right)}{D_{v}{}^{t}\left(x_{i}{}^{t},y_{i}{}^{t}\right)}\times \left[\frac{D_{v}{}^{t}\left(x_{i}{}^{t},y_{i}{}^{t}\right)}{D_{c}{}^{t}\left(x_{i}{}^{t},y_{i}{}^{t}\right)}/\frac{D_{v}{}^{t+1}\left(x_{i}{}^{t+1},y_{i}{}^{t+1}\right)}{D_{c}{}^{t+1}\left(x_{i}{}^{t+1},y_{i}{}^{t+1}\right)}\right]\times \left[\frac{D_{c}{}^{t}\left(x_{i}{}^{t},y_{i}{}^{t}\right)}{D_{c}{}^{t+1}\left(x_{i}{}^{t},y_{i}{}^{t}\right)}\times \frac{D_{c}{}^{t}\left(x_{i}{}^{t+1},y_{i}{}^{t+1}\right)}{D_{c}{}^{t+1}\left(x_{i}{}^{t+1},y_{i}{}^{t+1}\right)}\right] \end{array}$$ (11)

Eq (11) changes the original assumption that the scale remuneration is fixed and is suitable when the return of scale is changeable. This function further divides the comprehensive technical efficiency change (*CTEC*) into pure technical efficiency change (*PTEC*) and scale efficiency change (*SEC*) [41]. Subscript v shows the circumstance of changeable return of scale, whereas subscript c shows the circumstance of the eternal return of scale. The first part of Eq (11) shows the PTEC change. The second part is the change of *SEC*, whereas the third part is the same as Eq (10), which shows the *CTEC* change.

The Malmquist index method uses multiple input and output variables to analyze efficiency. It divides the cause of productivity change into technology change (*TC*) and *CTEC*, whereas *CTEC* can be subdivided into *PTEC* and *SEC*. Thus, the Malmquist index can be shown as: *ML* = *CTEC* × *TC* = *PTEC* × *SEC* × *TC*.

### Data and variables

#### Agricultural carbon emissions from carbon sources

Agriculture has a general and narrow definition. From a broad perspective, agriculture contains five kinds of industrial forms: planting, forestry, animal husbandry, fishery, and sideline industry, whereas the narrow sense refers to only the planting industry. The sources of agricultural carbon emissions can be subdivided into three sections. The first section includes the carbon emissions that are caused by agricultural land use, including chemical fertilizers, pesticides, and agricultural film. Carbon emissions from the use of diesel by agricultural machinery, the loss of organic carbon produced by soil destruction when tilling the soil, and the carbon release from the indirect consumption of fossil fuels during the irrigation process are all included in this section. Second is the production of methane and other greenhouse gases during rice growth. The third is carbon emissions from ruminant farming, including methane production from intestinal fermentation and excrement management. Agricultural carbon emissions are the total amount of carbon emissions that are produced by various types of carbon sources. According to the IPCC and existing literature, the carbon emission coefficients of various agricultural carbon sources are shown in Table 1.

**Table 1:** Carbon emission coefficients of various agricultural carbon sources.

Farming Land Utilization and Rice	Coefficient of Carbon Emission	Reference	Ruminant	Intestinal Fermentation	Feces Management	Reference
**Chemical fertilizer**	0.8956 kg(C)·kg^−1^	West and Marland, Oak Ridge National Laboratory [51]	Cow	416.02kg(C)·head^−1^·year^−1^	122.76kg(C)·head^−1^·year^−1^	IPCC [53]
**Pesticide**	4.9341kg (C)·kg^−1^		Buffalo	375.10kg(C)·head^−1^·year^−1^	13.64kg(C)·head^−1^·year^−1^	
**Agricultural film**	5.18kg (C)·kg^−1^	Agricultural resources and ecological, environment institute of Nanjing Agricultural University [52]	Other Cattle	320.54kg(C)·head^−1^·year^−1^	6.82kg(C)·head^−1^·year^−1^	
**Diesel**	0.5927kg (C)·kg^−1^	IPCC [53]	Horse	122.76kg(C)·head^−1^·year^−1^	11.18kg(C)·head^−1^·year^−1^	
**Irrigation**	266.48kg (C)·hm^−2^	West and Marland [51]	Donkey	68.20kg(C)·head^−1^·year^−1^	6.14kg(C)·head^−1^·year^−1^	
**Soil destruction**	312.60Kg(C)·hm^−2^	College of Biology and Technology of China Agricultural University [54]	Mule	68.20kg(C)·head^−1^·year^−1^	6.14kg(C)·head^−1^·year^−1^	
**Rice growth**	49.57kg (C)·hm^−2^	Wang et al. [55]	Pig	6.82kg(C)·head^−1^·year^−1^	27.28kg(C)·head^−1^·year^−1z^	
			Goat	34.10kg(C)·head^−1^·year^−1^	1.16kg(C)·head^−1^·year^−1^	
			Sheep	34.10kg(C)·head^−1^·year^−1^	1.02kg(C)·head^−1^·year^−1^	

According to Table 1, the total carbon emissions mainly include three parts. First, the carbon emissions of chemical fertilizers, pesticides, agricultural film and diesel oil are the product of the usage amount and carbon emission coefficient. The formula for calculating carbon emissions from agricultural land use is as follows: C_1_ = fertilizer use*0.8956 + pesticide use*4.9341 + agricultural film use*5.18 + diesel oil use* 0.5927. Second, the carbon emissions of agricultural irrigation, tillage and rice are the product of area and carbon emission coefficient. The formula for calculating carbon emissions from rice planting is as follows: C_2_ = irrigation area * 266.48 +soil destruction area * 312.6 +rice planting area* 49.57. Then, the carbon emissions of ruminants are divided into two parts, where one is the product of the number of animals and the coefficient of intestinal fermentation and the other is the product of the number of animals and the coefficient of fecal management. The formulas for calculating carbon emissions from ruminant farming are as follows: C_3_ = cow number* (416.02+122.76) + buffalo number* (375.1+13.64) +other cattle number* (320.54+6.82) + horse number *(122.76+11.18) +donkey number*(68.2+6.14) + mule number *(68.2+6.14) +pig number (6.82+27.28) +goat number *(34.1+1.16) +sheep number *(34.1+1.02). Therefore, the total carbon emissions from carbon sources are C = C_1_+ C_2_+ C_3_.

#### Selection of input and output indicators

According to previous research [3,7,11,15,19] and based on traditional macroeconomic theory, the input variables of the agricultural carbon emission efficiency of the provinces and cities along the B&R can be summarized with the following four items: (1) labor input, measured by the number of agricultural workers; (2) land input, measured by the cultivated land area; (3) capital investment, estimated by the annual investment in agricultural fixed assets and the PIM index that is used to convert the flow index into the stock index; and (4) input of agricultural material, which contains fertilizer input, diesel fuel, agricultural film input, and pesticide input. Fertilizer input is measured by the actual amount of fertilizer application, including nitrogen, phosphorus, potassium, and compound fertilizers. Diesel fuel is measured by the annual use of diesel in the agricultural sector. Agricultural film input is measured by the annual consumption of the membrane. Pesticide input is measured by the annual use of pesticides.

The total output values of agriculture, forestry, animal husbandry and fisheries were selected as the expected outputs and agricultural carbon emissions as the undesirable outputs. To eliminate the interference of prices, this study calculated the total output values of agriculture, forestry, animal husbandry, and fisheries in 2006. When using the DEA model to evaluate the environmental efficiency, the efficiency is usually presented in the form of the evaluation index. The greater the index value, the higher the efficiency level. Therefore, higher efficiency requires less input and more output. The input and output indicators can be seen in Table 2.

**Table 2:** Input and output indicators of agricultural carbon emissions.

Category of Indicator	1st Tier Indicator	2nd Tier Indicator
**Input**	Labor input	Number of agricultural workers
	Land input	Cultivated land area
	Capital investment	Annual investment in fixed agricultural assets
	Agricultural materials input	Amount of fertilizer
		Amount of diesel
		Amount of agricultural film
		Amount of pesticides
**Output**	Expected output	The total output value of agriculture, forestry, animal husbandry and fishery
	Undesirable output	Agricultural carbon emissions

The DEA model requires both the input and output data of each decision unit to be positive. As a negative output index, carbon dioxide emissions cannot meet the operating conditions of the DEA model, and the selection of the BCC model results in evaluation invalidity. Thus, CO_2_ emission indicators must be transformed accordingly. The current applicable methods are curved measure evaluation, pollutant input treatment, data conversion function processing, and the directional distance function. The research selected the data conversion function method [56], as this method is regarded as one of the most satisfactory efficiency assessment methods and includes different types of negative outputs and linear and nonlinear data conversions. Since the linear data conversion method is more advantageous in the model with variable returns to scale analysis (VRS), this method is used to transform the environmental pollutants index data. It can be measured as: Y^’^_i_ = -Y+C, where C represents a large vector to ensure that all converted output data are positive.

#### Data sources

The data for the variables were obtained from the Chinese Rural Statistical Yearbook (2007–2016) [57], the Annals of China’s Environmental Statistics (2007–2016) [58], the National Bureau of Statistics of China [59], and the Chinese economic database [60].

## Results

Based on the steps, the descriptive statistical results of variables are calculated and listed in Table 3.

**Table 3:** Descriptive statistical results of variables.

Category	Variable	Sample number	Average	Standard deviation	Minimum	Maximum
Input	Agricultural workers (10 thousand people)	180	619.35	496.1	12.84	1677
	cultivated land area (million hectare)	180	393.83	356.61	18.76	1586.59
	Investment in agricultural fixed assets (100 million yuan)	180	434.82	351.33	3.1	1701.81
	Input of agricultural materials (10 thousand tons)	180	135.69	86.46	4.57	279.26
Output	GDP (100 million yuan)	180	1026.89	677.45	54.33	2706.65
	Agricultural carbon emissions (10 thousand tons)	180	999.69	386.26	149.19	1598.82

### Measurement of agricultural carbon emission efficiency based on BCC model

With the use of DEAP 2.0 (the Computer Vision and Systems Laboratory at University Laval, Quebec City, Canada) and the BCC model, this paper evaluated the agricultural carbon emission efficiency of 18 key provinces and cities (there are 16 provinces and two municipalities in total; for ease of reference, the paper refers to them all as provinces) along the B&R. The empirical model determined the comprehensive technical efficiency, pure technical efficiency, and the change of economies of scale.

#### Comprehensive technical efficiency of agricultural carbon emissions

Among the three efficiency indicators, the comprehensive technical efficiency most directly reflects the efficiency values of the 18 provinces within 10 years, which were affected by both the pure technical efficiency and scale efficiency. Additionally, efficiency here is a relative measure (relative to other provinces) rather than an absolute one. Therefore, the paper selected the comprehensive technical efficiency value as a measurement, and the result is shown in Table 4.

**Table 4:** Comprehensive technical efficiency of agricultural carbon emissions of the key provinces along the Belt and Road (B&R) within 2006–2015.

Area	Province	2006	2007	2008	2009	2010	2011	2012	2013	2014	2015	Average
**The Silk Road Economic Belt**	**Xinjiang**	1.000	1.000	1.000	1.000	1.000	1.000	1.000	1.000	1.000	1.000	1.000
	**Shaanxi**	0.500	0.603	0.615	0.612	0.618	0.557	0.625	0.563	0.494	0.518	0.571
	**Gansu**	0.471	0.543	0.578	0.591	0.639	0.643	0.540	0.608	0.588	0.597	0.580
	**Ningxia**	0.691	0.736	0.751	0.780	0.814	0.814	0.839	0.826	0.789	0.789	0.783
	**Qinghai**	0.876	0.927	0.982	0.925	0.938	1.000	0.806	0.887	0.862	0.817	0.902
	**Inner Mongolia**	0.751	0.826	0.806	0.839	0.571	0.636	0.739	0.697	0.666	0.618	0.715
	**Heilongjiang**	0.707	0.874	0.918	0.862	0.855	0.850	1.000	1.000	1.000	1.000	0.907
	**Jilin**	0.798	0.871	0.855	0.866	0.664	0.676	0.789	0.713	0.705	0.722	0.766
	**Liaoning**	1.000	1.000	1.000	1.000	0.987	1.000	1.000	1.000	1.000	1.000	0.999
	**Guangxi**	0.692	0.801	0.791	0.745	0.701	0.650	0.662	0.612	0.578	0.580	0.681
	**Yunnan**	0.567	0.599	0.617	0.631	0.570	0.549	0.607	0.586	0.566	0.549	0.584
	**Tibet**	1.000	1.000	1.000	1.000	1.000	1.000	1.000	1.000	1.000	1.000	1.000
	**Chongqing**	0.614	0.605	0.602	0.570	0.513	0.487	0.493	0.487	0.475	0.492	0.534
	**Avg**.	0.744	0.799	0.809	0.802	0.759	0.759	0.777	0.768	0.748	0.745	
**The 21st-Century Maritime Silk Road**	**Shanghai**	1.000	1.000	1.000	1.000	1.000	1.000	1.000	1.000	1.000	1.000	1.000
	**Fujian**	1.000	1.000	1.000	1.000	1.000	0.963	1.000	1.000	1.000	1.000	0.996
	**Guangdong**	1.000	1.000	1.000	1.000	1.000	0.908	0.945	0.946	0.960	0.975	0.973
	**Zhejiang**	1.000	1.000	1.000	1.000	1.000	1.000	1.000	1.000	1.000	1.000	1.000
	**Hainan**	1.000	1.000	1.000	1.000	1.000	1.000	1.000	1.000	1.000	1.000	1.000
	**Avg**.	1.000	1.000	1.000	1.000	1.000	0.974	0.989	0.989	0.992	0.995	
**B&R Average**		0.815	0.855	0.862	0.857	0.826	0.819	0.836	0.829	0.816	0.814	

Table 3 shows that the agricultural carbon emission efficiencies of the five provinces in Xinjiang, Tibet, Shanghai, Zhejiang and Hainan have long reached the effective frontier of comprehensive technology. There are five provinces with high efficiencies (0.8 < *x* < 1): Qinghai, Heilongjiang, Liaoning, Fujian and Guangdong. The remaining eight provinces are classified in the middle level: Shaanxi, Gansu, Ningxia, Inner Mongolia, Jilin, Guangxi, Yunnan, and Chongqing. Forty-five percent of the provinces remain in the medium efficiency level, which highlights the importance of improving carbon emission efficiency. Carbon emission reduction strategies need to be further implemented.

Second, from the fluctuation of the comprehensive technical efficiency of each province over the last 10 years, except for the five provinces of Xinjiang, Tibet, Shanghai, Zhejiang and Hainan which have already reached the effective frontier, the fluctuation range of Shaanxi, Gansu, Ningxia, Qinghai, Liaoning, Chongqing, Fujian and Guangdong is basically between 0.1 and 0.2, which is a small range. Additionally, the fluctuation range of efficiency in Inner Mongolia, Heilongjiang, Jilin and Guangxi is between 0.2 and 0.3. Since the launch of China’s the Belt and Road (B&R) initiative in 2013, the carbon emission efficiency of all other provinces has not achieved sustained growth except for Guangdong.

#### Pure technical efficiency and economies of scale of agricultural carbon emission

The comprehensive technical efficiency is affected by both the pure technical efficiency and scale efficiency, which can be calculated as: comprehensive technical efficiency = pure technical efficiency × scale efficiency. The paper further discusses how the latter two efficiencies affect the comprehensive technical efficiency of each province. The details are provided in Table 5.

**Table 5:** Average value of the efficiencies of key provinces along the B&R, 2006–2015.

Regions	Provinces	Comprehensive technical efficiency	Pure technical efficiency	Scale efficiency
**The Silk Road Economic Belt**	**Xinjiang**	1.000	1.000	1.000
	**Shaanxi**	0.571	0.729	0.789
	**Gansu**	0.580	0.609	0.953
	**Ningxia**	0.783	0.832	0.941
	**Qinghai**	0.902	0.922	0.978
	**Inner Mongolia**	0.715	0.794	0.917
	**Heilongjiang**	0.907	0.966	0.938
	**Jilin**	0.766	0.806	0.956
	**Liaoning**	0.999	1.000	0.999
	**Guangxi**	0.681	0.839	0.812
	**Yunnan**	0.584	0.676	0.865
	**Tibet**	1.000	1.000	1.000
	**Chongqing**	0.534	0.546	0.980
**The 21st-Century Maritime Silk Road**	**Shanghai**	1.000	1.000	1.000
	**Fujian**	0.996	1.000	0.996
	**Guangdong**	0.973	1.000	0.973
	**Zhejiang**	1.000	1.000	1.000
	**Hainan**	1.000	1.000	1.000

Table 4 shows that five provinces—Xinjiang, Tibet, Shanghai, Zhejiang, and Hainan—have long reached comprehensive technical efficiency. Their pure technical efficiency and efficiency are both 1, proving that they have reached the frontiers from both perspectives. Shaanxi, Gansu, Ningxia, Qinghai, Inner Mongolia, Jilin, Yunnan, and Chongqing are the provinces whose pure technical efficiencies are less than their scale efficiencies. For these provinces, the lag in carbon emission reduction technology is restricting the efficiency of their agricultural carbon emissions. The provinces with lower scale efficiency than pure technical efficiency are Heilongjiang, Guangxi, and Guangdong, showing that their scale of resource allocation is unreasonable and that appropriate adjustments should be undertaken.

#### Increase/decrease in economies of scale of agricultural carbon emission

For the majority of provinces, the lag in technology is the key factor restricting the efficiency of their agricultural carbon emission. For the scale efficiency indicator, only five provinces reached the best production frontier, and considerable improvements are needed in the other 13 provinces, indicating that the allocation of the input resources needs to be improved in those 13 provinces. Therefore, the paper further investigated the increase/decrease in the scale remuneration of the agricultural carbon emissions from 2006 to 2015 in different provinces (Table 6).

**Table 6:** Increase/decrease in economies of scale of agricultural carbon emissions of key provinces along the B&R, 2006–2015.

Regions	Provinces	2006	2007	2008	2009	2010	2011	2012	2013	2014	2015
**The Silk Road Economic Belt**	**Xinjiang**	—	—	—	—	—	—	—	—	—	—
	**Shaanxi**	drs	drs	drs	drs	drs	drs	drs	drs	drs	drs
	**Gansu**	drs	drs	drs	drs	drs	drs	drs	drs	drs	drs
	**Ningxia**	drs	drs	irs	drs	drs	irs	irs	irs	irs	irs
	**Qinghai**	drs	drs	drs	irs	irs	—	irs	irs	irs	irs
	**Inner Mongolia**	drs	drs	drs	drs	drs	drs	drs	drs	drs	irs
	**Heilongjiang**	drs	drs	drs	drs	drs	drs	—	—	—	—
	**Jilin**	drs	drs	drs	drs	drs	drs	drs	drs	drs	drs
	**Liaoning**	—	—	—	—	drs	—	—	—	—	—
	**Guangxi**	drs	drs	drs	drs	drs	drs	drs	drs	drs	drs
	**Yunnan**	drs	drs	drs	drs	drs	drs	drs	drs	drs	drs
	**Tibet**	—	—	—	—	—	—	—	—	—	—
	**Chongqing**	irs	drs	drs	drs	drs	—	drs	drs	irs	drs
**The 21st-Century Maritime Silk Road**	**Shanghai**	—	—	—	—	—	—	—	—	—	—
	**Fujian**	—	—	—	—	—	drs	—	—	—	—
	**Guangdong**	—	—	—	—	—	drs	drs	drs	drs	drs
	**Zhejiang**	—	—	—	—	—	—	—	—	—	—
	**Hainan**	—	—	—	—	—	—	—	—	—	—

Notes: drs means decreasing scale remuneration;

“—”means no change of scale remuneration;

“irs” means increasing scale remuneration.

Shanxi, Gansu, Jilin, Guangxi, and Yunnan demonstrated a long-term decreasing trend. In these areas, radial movement should be reduced, and the rational allocation of resources should be optimized to improve the efficiency of agricultural carbon emissions. Similar strategies also apply to Inner Mongolia and Chongqing. The four efficient provinces of Guangdong, Fujian Liaoning, and Heilongjiang have some years of diminishing returns on the scale. Thus, governments should strive to improve scale efficiency and achieve the frontier of production in comprehensive technical efficiency. For Ningxia and Qinghai, which maintained increasing returns to scale in most of the years, a possible strategy involves increasing the input materials to improve carbon emission efficiency. For Xinjiang, Tibet, Shanghai, Zhejiang, and Hainan, the scale of remuneration remained unchanged for 10 years.

#### Regional comparison of agricultural carbon emissions

**Fig 1:**
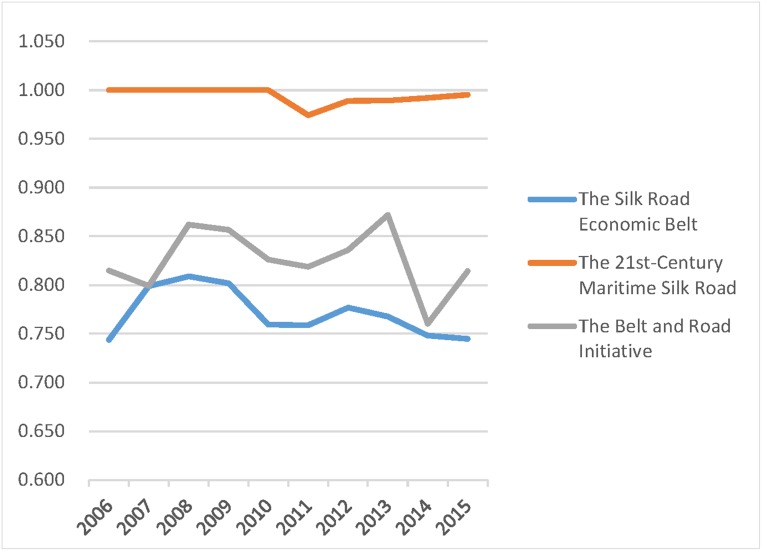
Comprehensive technical efficiency of different regions, 2006–2015.

**Fig 2:**
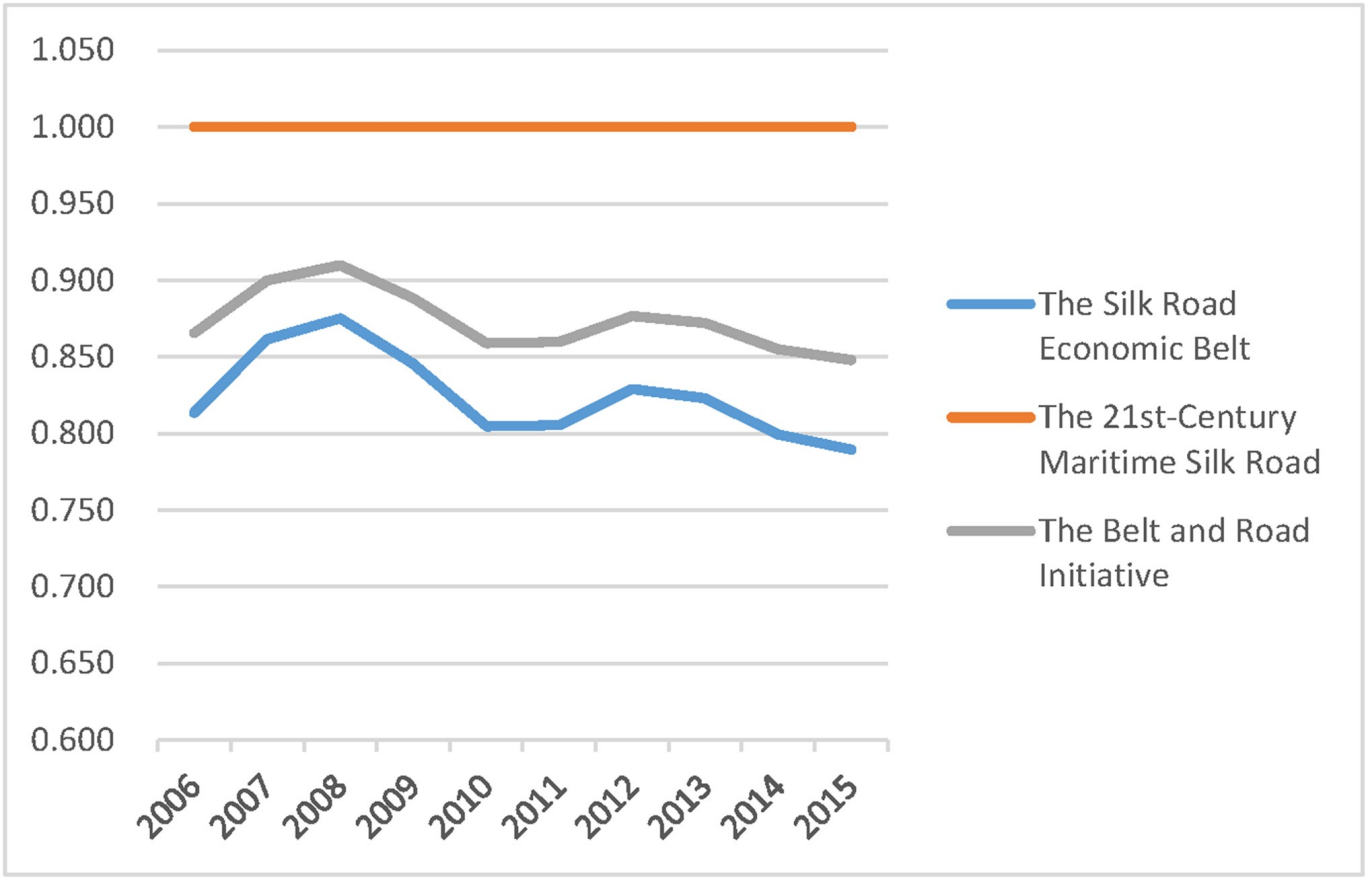
Pure technical efficiency of different regions, 2006–2015.

**Fig 3:**
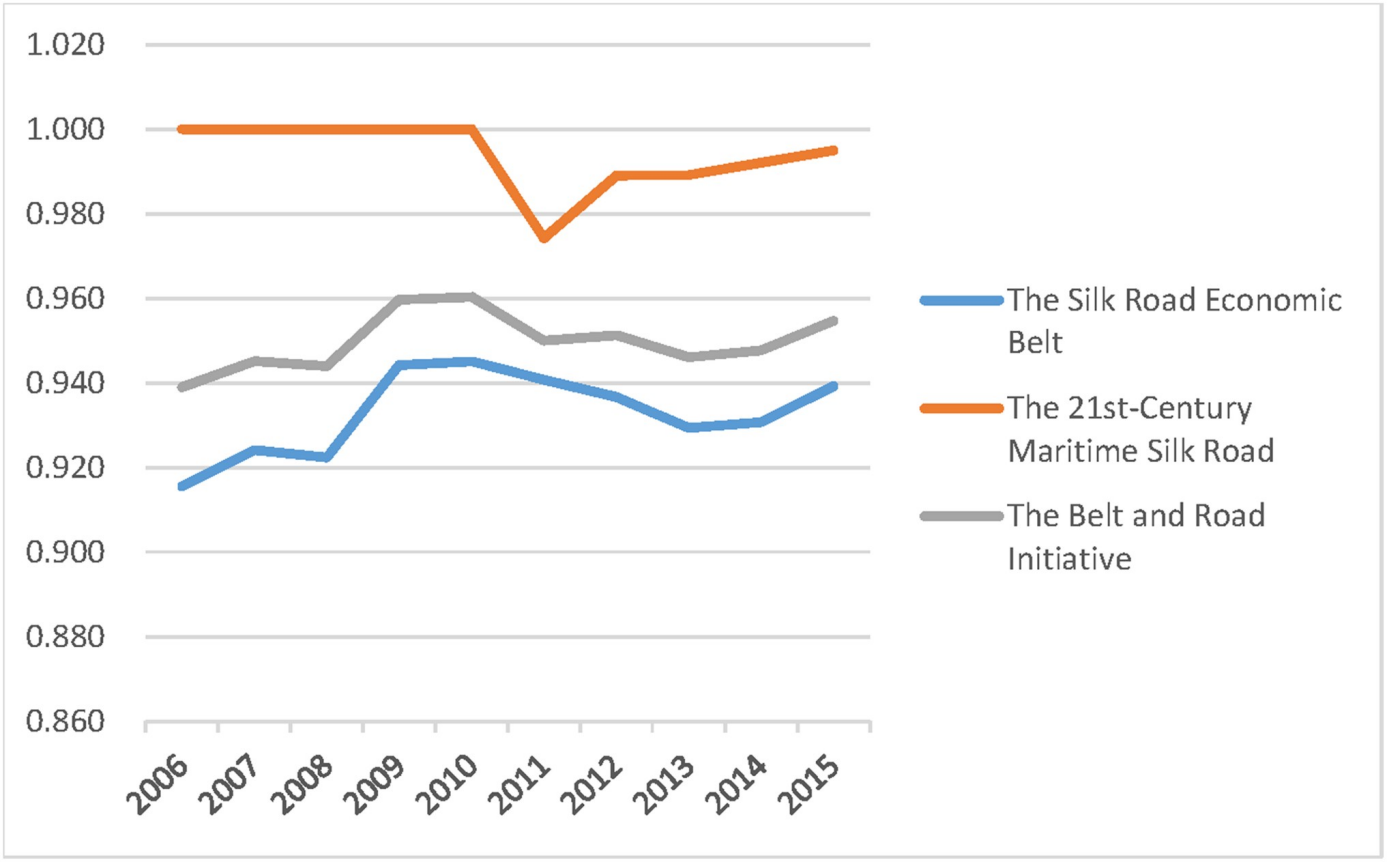
Scale efficiency of different regions, 2006–2015.

The Belt and Road Initiative is divided into the 21st-Century Maritime Silk Road and The Silk Road Economic Belt. Each contains different provinces, and a detailed classification is outlined in Tables 4–6. The paper further compared the three average values of the efficiencies in those regions to provide useful advice on how to strengthen exchange and cooperation between regions. The results are shown in Figs 1–3.

The above figures show that for any of the three efficiencies, the overall efficiency of the B&R always falls between the higher values for the 21st-Century Maritime Silk Road provinces and the lower values of The Silk Road Economic Belt provinces. Therefore, the governance of agricultural carbon emissions should focus on the provinces along The Silk Road Economic Belt. Those provinces along the 21st-Century Maritime Silk Road benefit from a small proportion of agricultural industry, fewer carbon emissions from agriculture, high total economic volume, and massive investment in environmental management. They have achieved an ideal state of agricultural carbon emissions. It is highly recommended that those provinces should strengthen communication and cooperation with provinces along the Silk Road Economic Belt, providing experience and financial support for carbon emission reduction.

### Dynamic evolution analysis of agricultural carbon emission efficiency based on the Malmquist index model

With the use of the DEA-Malmquist index, the paper could test the dynamic change in the agricultural carbon emission efficiency in various provinces. In the process of dynamic evolution, it can determine whether the efficiency has been promoted since the B&R initiative in 2013 to measure the impact of the initiative on China’s environmental protection scheme.

#### Temporal dynamic evolution of agricultural carbon emission efficiency

Table 7 shows that the total factor productivity of the key provinces in China fluctuated during the period of 2006 to 2015. The main trend was downward, except for an increase of 6.5% from 2010 to 2011. Even after 2013, the total factor productivity still demonstrated a diminishing trend, which further indicates that the B&R initiative has not improved China’s carbon emission efficiency. From the decomposition of the Malmquist index, the reduction in productivity was mainly due to the mobile efficiency of the production frontier or technological change. During the descending periods, this change led to a 5.45% average decrease in the total factor productivity. Thus, the paper concludes that the reduction of the technology utilization level is the critical issue restricting the efficiency of agricultural carbon emissions. Even after the introduction of the B&R initiative in 2013, technology still causes a downward trend in total factor productivity, and there was no substantial change thereafter.

**Table 7:** Temporal dynamic evolution in agricultural carbon emission efficiency of the provinces along B&R, 2006–2015.

Time	Comprehensive Technical Efficiency Change	Technical Change	Pure Technical Efficiency Change	Scale Efficiency Change	Malmquist
**2006–2007**	1.059	0.915	1.050	1.008	0.969
**2007–2008**	1.010	0.981	1.012	0.998	0.991
**2008–2009**	0.994	0.906	0.977	1.017	0.901
**2009–2010**	0.957	0.957	0.957	1.000	0.916
**2010–2011**	0.996	1.069	1.000	0.996	1.065
**2011–2012**	1.017	0.896	1.024	0.993	0.911
**2012–2013**	0.989	1.005	0.995	0.994	0.994
**2013–2014**	0.978	0.986	0.977	1.001	0.965
**2014–2015**	0.999	0.960	0.990	1.009	0.959
**2006–2015**	1.000	0.963	0.998	1.002	0.962

In addition to the temporal analysis of the dynamic changes in the agricultural carbon emission efficiency of the provinces, the paper also analyzed the specific conditions of each province, as shown in Table 8. First, the results revealed that the agricultural carbon emission efficiencies of the key provinces were trending downward. Only 3 of the 18 provinces, Ningxia, Heilongjiang, and Shanghai, had a Malmquist greater than 1. Second, the technical improvement index for all the other provinces was less than 1, except for Shanghai, which verifies that the lag in technology is the key factor in reducing agricultural carbon emissions.

**Table 8:** Dynamic evolution of agricultural carbon emission efficiency of each province along the B&R, 2006–2015.

Regions	Provinces	Comprehensive Technical Efficiency Change	Technical Change	Pure Technical Efficiency Change	Scale Efficiency Change	Malmquist
**The Silk Road Economic Belt**	**Xinjiang**	1.000	0.982	1.000	1.000	0.982
	**Shaanxi**	1.004	0.925	1.038	0.968	0.928
	**Gansu**	1.027	0.958	1.023	1.003	0.984
	**Ningxia**	1.015	0.988	0.997	1.018	1.003
	**Qinghai**	0.992	0.982	0.997	0.996	0.975
	**Inner Mongolia**	0.979	0.979	0.949	1.031	0.958
	**Heilongjiang**	1.039	0.992	1.020	1.019	1.031
	**Jilin**	0.989	0.980	0.971	1.018	0.970
	**Liaoning**	1.000	0.978	1.000	1.000	0.978
	**Guangxi**	0.981	0.930	0.993	0.987	0.912
	**Yunnan**	0.996	0.955	1.000	0.996	0.952
	**Tibet**	1.000	0.873	1.000	1.000	0.873
	**Chongqing**	0.976	0.958	0.976	1.000	0.935
	**Avg**.	1.000	0.960	0.997	1.003	0.960
**The 21st-Century Maritime Silk Road**	**Shanghai**	1.000	1.002	1.000	1.000	1.002
	**Fujian**	1.000	0.978	1.000	1.000	0.978
	**Guangdong**	0.997	0.938	1.000	0.997	0.935
	**Zhejiang**	1.000	0.970	1.000	1.000	0.970
	**Hainan**	1.000	0.965	1.000	1.000	0.965
	**Avg**.	0.999	0.971	1.000	0.999	0.970

#### Spatial dynamic evolution of agricultural carbon emission efficiency

The paper also discussed the spatial difference and evolution of the agricultural carbon emission efficiency of key provinces along the B&R with the use of the Malmquist index and ArcGIS (Environmental Systems Research Institute, RedLands, and The United States). The spatial distribution was measured in different periods. The paper selected the years 2007, 2011, and 2015 to observe the spatial differences in agricultural carbon emissions at different time points and then discussed their evolution characteristics.

**Fig 4:**
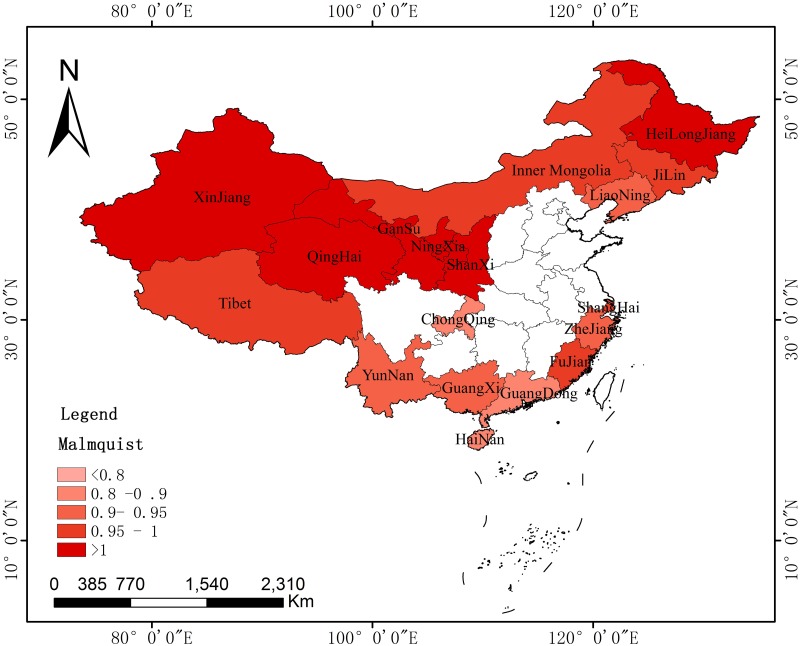
Spatial distributions of the Malmquist index of key provinces along the B&R in 2007.

**Fig 5:**
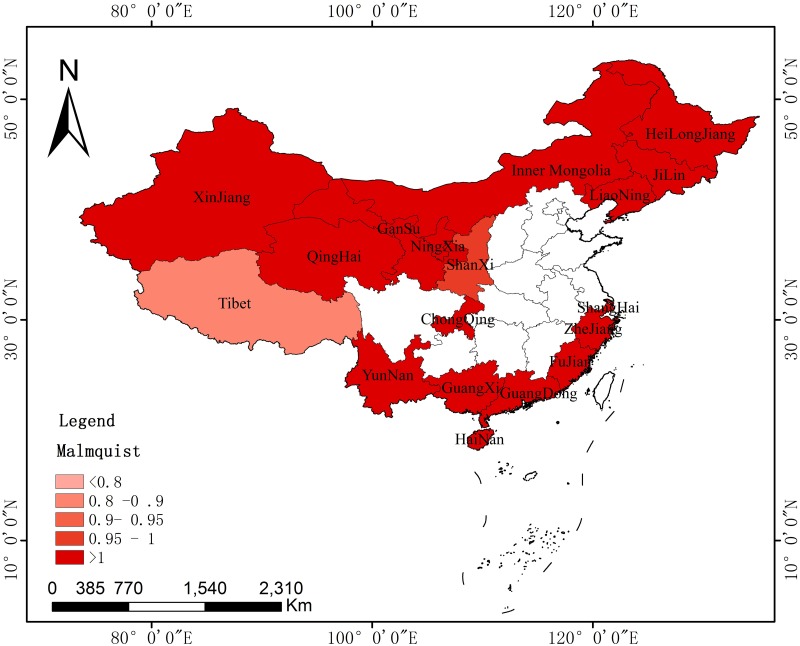
Spatial distributions of the Malmquist index of key provinces along the B&R in 2011.

**Fig 6:**
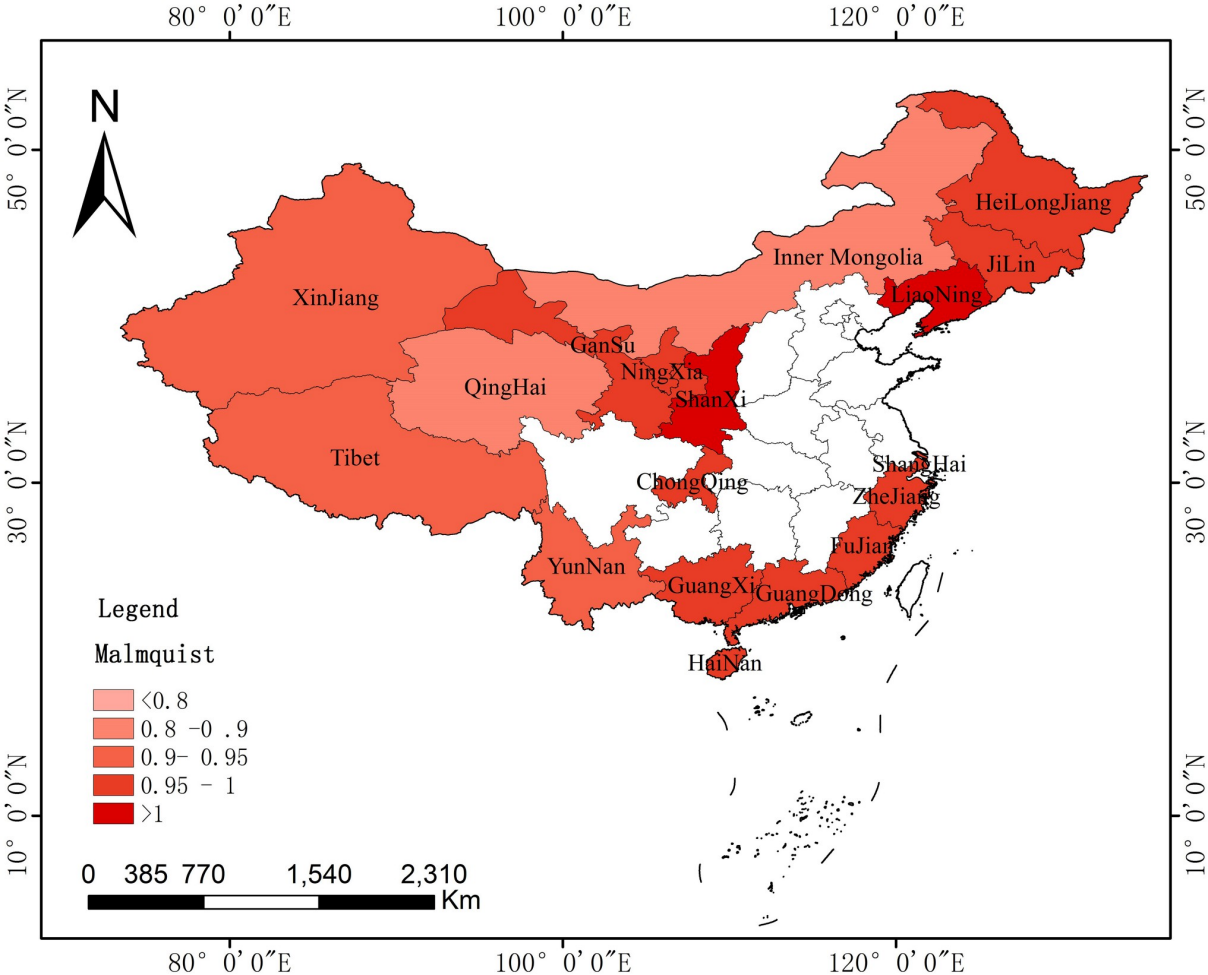
Spatial distributions of the Malmquist index of key provinces along the B&R in 2015.

Figs 4–6 reflect the spatial evolution at three key time points from 2006 to 2015. It regards 2011 as the best time point, as the Malmquist of most provinces (16) was greater than 1, with only one in the 0.9–0.95 and another in the 0.8–0.9 range. In 2007, the Malmquist index increased in six provinces, with a value larger than 1. Five of the other provinces remained within 0.95–1, while the others had smaller values. For 2015, the performance was even more unsatisfactory, with two provinces experiencing an increase in the Malmquist index. The other cities were categorized into groups of 0.95–1, 0.9–0.95, 0.8–0.9, or less than 0.8. This difference further illustrates the conclusion provided in the previous section.

## Discussions and conclusions

Exploring the efficiency is the basic premise in further research on China’s agricultural carbon emissions [61], which helps explain agricultural “water-land-energy-carbon” (WLEC) nexus and improve the efficiency of agricultural resources use [62]. Based on the panel data of 18 key provinces and cities along the B&R between 2006 and 2015, the paper evaluated agricultural carbon emissions efficiency using the DEA-BCC model and further explored their dynamic evolution trend with the DEA-Malmquist model. The results showed that: (1) the level of agricultural carbon emissions in various provinces was uneven. Xinjiang, Tibet, Shanghai, Zhejiang, and Hainan were at the forefront of agricultural carbon emissions, with the five provinces of Qinghai, Heilongjiang, Liaoning, Fujian, and Guangdong being highly efficient. The remaining eight provinces demonstrated medium efficiency. For most provinces, the efficiency of agricultural carbon emissions must be improved. (2) Technical efficiency is the key factor that restricts the promotion of comprehensive efficiency of agricultural carbon. (3) Although the impact of scale efficiency on comprehensive efficiency is less than that of technical efficiency, except for five provinces at the frontier of production, the scale efficiency of the other provinces requires improvement. Through the increase or decrease in scale remuneration, most provinces demonstrated downward trends in their economies of scale, implying that their investment is too redundant, and that the allocation of resources is not reasonable. (4) The efficiency level of agricultural carbon emissions presented significant regional differentiation among the areas, with those along the 21st-Century Maritime Silk Road being much higher than those included in the Silk Road Economic Belt. (5) According to dynamic evolution analysis, the total factor productivity still demonstrated a diminishing trend. Therefore, although the B&R Initiative has promoted the exchange and development of China with the surrounding countries in the economic, cultural, and political arenas, the initiative has contributed little to the environmental amelioration of our country, which is contrary to the concept of sustainable development, which should be seriously considered by the Chinese government.

From the above research results, the paper provides some suggestions to effectively enhance the efficiency of agricultural carbon emissions in China. (1) The government should optimize the agricultural industrial structure and accelerate agricultural modernization in the Belt and Road region. (2) A low-carbon agricultural development mechanism should be constructed with the basis of the modern agricultural industrial technology system, and scientific research should be integrated and applied to the whole process of agricultural production. (3) Each province should increase their investment in low-carbon agriculture and establish a complete set of low-carbon agricultural ecological compensation technology system to play China's model as a leading role in low-carbon agriculture along the Belt and the Road. (4) Due to the significant regional differences in agricultural carbon emission efficiencies, the focus of agricultural energy conservation and emission reduction work should be on striving to realize the transformation of agricultural production to a low-carbon direction in the Silk Road Economic Belt areas.

There are also some deficiencies in this paper in the availability of data and deficiencies at the author's own level, such as the lack of deep excavation about the causes and influencing factors for the differences in regional agricultural carbon emissions and the agricultural carbon emission efficiencies of various countries or regions along the Belt and Road region. Then, the cooperation and incentive mechanisms of agricultural carbon emission reduction are not involved. Next, the authors will further research these problems that urgently need to be solved. There are also some deficiencies in this research in the availability of data and the deficiencies at the author's own level, such as the lack of deep excavation about the causes and influencing factors in the differences in regional agricultural carbon emissions and the agricultural carbon emission efficiencies of various countries or regions along the Belt and Road region. Then, the cooperation and incentive mechanisms of agricultural carbon emission reduction are not involved. Next, the authors will further research these problems that urgently need to be solved.
